# Homoplasy as an Auxiliary Criterion for Species Delimitation

**DOI:** 10.3390/microorganisms9020273

**Published:** 2021-01-28

**Authors:** Angela Conti, Debora Casagrande Pierantoni, Vincent Robert, Gianluigi Cardinali, Laura Corte

**Affiliations:** 1 Department of Pharmaceutical Sciences, University of Perugia, 06121 Perugia, Italy; angela.conti@studenti.unipg.it (A.C.); deboracasagrandepierantoni@gmail.com (D.C.P.); laura.corte@unipg.it (L.C.); 2 Westerdjik Institute for Biodiversity, Uppsalalaan 8, 3584 CT Utrecht, The Netherlands; v.robert@wi.knaw.nl; 3 CEMIN Excellence Research Centre, via Elce di Sotto 8, 06123 Perugia, Italy

**Keywords:** homoplasy, consistency, species, yeast, LSU, ITS, HI, CI, delimitation, HGT

## Abstract

Homoplasy is a sort of noise in phylogenetic reconstructions, due to the accumulation of backmutations, convergent evolution and horizontal gene transfer (HGT), which is considered the major trigger of homoplasy in microorganism for its massive presence. It is also known that homoplasy increases with the complexity of the tree with both real and simulated data. In this paper, we analyzed the variation of homoplasy with the two widely used taxonomic markers *ITS* and *LSU* in four taxonomic models characterized by differences in the intra-specific distances. An algorithm (HomoDist) was developed to analyze the homoplasy index (HI) variation upon addition of a single element (strain or species) in increasing distance from a starting element. This algorithm allows to follow changes of the consistency index (CI), complementary to the HI, with the increase of the number of taxa and with the increase of the distance among elements. Results show that homoplasy increases—as expected—with the number of taxa, but also as a function of the overall distance among species, often with an almost linear relationship between distance and HI. No HI change was observed in trees with few taxa spanning through short distances, indicating that this noise is not prohibitive in this context, although the analysis of the ratio between HI and distance can be recommended as a criterion for tree acceptance. The absence of large changes of the HI within the species, and its increase when new species are added by HomoDist, suggest that homoplasy variation can be used as an auxiliary test in distance-based species delimitation with any type of marker.

## 1. Introduction

The word homoplasy was used for the first time by the British zoologist Lankester in 1870 to dissect the general world “homology” in “homogeny” and “homoplasy,” using the following definition: “homoplasy includes all cases of close resemblance of form which are not traceable to homogeny” [1]. Whilst the term homogeny has turned to be homology in current biological semantics, the term homoplasy survived and was taken over by Willy Henning with the same specific meaning given by Lankester [2]. Current phylogenetics refer now to homoplasy as the situation in which traits are common to taxa not sharing a common ancestry, that can be caused by convergent evolution, reversal to ancestral trait and horizontal gene transfer (HGT) [3,4]. Homoplasy is crucial in systematics for two major reasons: (i) a high level of homoplasy decreases the reliability of the obtained dendrogram [5], (ii) homoplasy is an indirect measure of the “species semipermeable boundaries” and then, indirectly, the level of gene flow between populations [6]. The first aspect relates to the fact that homoplasy is the phylogenetic noise hampering the search of a consistent tree [7] and influencing clade support metrics as the bootstrap [7], although homoplastic sites introduce more information on the phylogenetic structure under study [8]. The need of homoplasy quantification as an *a posteriori* control comes directly from the first Henning’s auxiliary principle that assumes homology at the basis of similarities among characters, in the absence of contrary evidence [2,9]. The quantification of homoplasy was introduced by Kluge and Parris in 1969 with the consistency index (CI) complementary to the homoplasy index (HI) [10]. Other metrics exist such as the retention index (RI) [11,12] and the rescaled consistency index (RCI) [13], with slight differences and a common aspect: when these indexes are 0 or close to 0 the homoplasy is high and all characters derive from apparent homology, i.e., homoplasy. In contrast, when these quality indexes are high up to 1, all character states are due to synapomorphy. It was noted that the homoplasy measures increase with the number of taxa more than with the number of characters [14,15].

The second relevant aspect of homoplasy derives from the fact that horizontal gene transfer (HGT) is probably the most important component of homoplasy both in prokaryotes and some eukaryotes such as fungi, implying that genetic exchange can occur among microbial species [16]. The concept that microbial species are not genetically impermeable is somehow in contrast with the well-known biological species concept [17], although Ernst Mayr himself was clear in delimiting the application of this concept to species that reproduce only sexually [18]. Yeasts have the peculiar characteristic of being able to reproduce both sexually and asexually [19]. Thus, yeasts combine traits proper to both eukaryote and prokaryota. For this reason, together with the ease of cultivation and manipulation, they are considered consolidated model in genetics, phylogenetics and taxonomy [20]. An extensive critique to the various species concepts, reviewed from different points of view support the idea that the microbial species cannot be considered a group of organisms without anything in common with other species [16,21]. Moreover, the evidence is mounting that introgressions are common in microorganisms and that species hybrids are frequently discovered in industrial and natural environments [22,23]. These evidences recently led to take advantage of homoplasy as part of an index indicating the presence of gene flow at the genomic level [6]. From the computational viewpoint, the massive presence of HGT in prokaryotes has posed the technical question on the possibility to continue using dendrograms answered in this same Special Issue by a vast analysis on the necessity of networks and dendrograms [3].

This overview on the role and importance of homoplasy underlines that this is a key phenomenon in evolution and speciation and therefore on the various approaches to define or delimit species with taxonomic, genetic and phylogenetic purposes. The many approaches to species delimitation can be grouped into three major categories: phylogeny (genealogical coherence), reproductive isolation and phenetics, i.e., phenotypic distinctiveness [24]. Reproductive isolation can only be used in some cases and almost never in absence of obligate sexual reproduction, furthermore, the biological species concept is not straightforward to apply for the species delimitation and rather hard in everyday identification practice. On the contrary, phylogenetics and phenetics are fully operational both in classification (i.e., when the species boundaries are defined) and in identification (i.e., when a strain is assigned to a described species). Both phenetics and phylogenetics (with the exclusion of parsimony) rely on distances among strains calculated with various algorithms based on the character states. In this context, the DNA encoding for the ribosomal RNA (rRNA) has found large application in both taxonomy and phylogeny [25,26,27,28]. Although the rDNA markers are relatively short in length, there is evidence of a relatively high level of homoplasy at least in eukaryotes [29], probably because of the internal heterogeneity among the several copies of these loci [30,31]. Even though species delimitation is moving rapidly to genomic based approaches [22,32,33,34], most of the everyday taxonomic practice makes use of one or few markers from these loci, that are also particularly abundant in reference databases. In this context, we took into consideration two loci to analyse the patterns of homoplasy variations in defined and well explored groups of yeasts.

Given the importance of homoplasy in defining the quality of the phylogenetic reconstructions and the mechanisms of speciation, the aim of this paper is to compare distances among strains and the corresponding homoplasy. The mechanism of this analysis is to aggregate strains to gradually increase the distances of the groups under study and to calculate the CI and other measures at every aggregation. The rationale is to compare the trend of homoplasy vs. distance when the analysis is carried out within known species and within genera containing several species.

## 2. Materials and Methods

### 2.1. Models Adopted for the Analysis

All the analyses were carried out considering organisms belonging to four different genera: *Candida, Debaryomyces, Kazachstania* and *Saccharomyces. ITS* and *LSU D1/D2* sequences (Table 1) of the species included in the study were retrieved from the public database YeastIP (http://genome.jouy.inra.fr/yeastip/), while the same marker sequences (Table S1) of the strains, chosen for the analysis, were obtained from the National Centre for Biotechnology Information (NCBI). For each model, the analyses were carried out on two different levels: the species level, for which only marker sequences of the type strains were considered, and strain level, in which *ITS* and *LSU D1/D2* sequences of five different organisms for each species under test were considered.

### 2.2. Alignment and Sequences Analysis

*ITS* and *LSU D1/D2* sequences were collected in two separate FASTA files, one for the analysis of the species using the type strains and one for the analysis with more strains per species. These sequences were aligned with the algorithm ClustalW in MEGA 7 [35].

The rDNA operon was considered as two independent markers as to avoid an over-estimation of distances due to the differences in the joining regions. The parameters chosen for the alignment were: Gap Opening Penalty 15 and Gap Extension Penalty 6.66 for both pairwise and multiple alignment, while transition weight was considered equal to 0.3. The initial and the final portion of the alignment were trimmed to have sequences of the same length.

### 2.3. HomoDist Algorithm

The rationale of the algorithm is that homoplasy variations can be analyzed by producing trees of increasing complexity spanning through increasingly larger distances. This algorithm, named *HomoDist*, was written as a simple R script and is available upon request. The algorithm starts by ordering the strains or, in general the taxa, in increasing order of distance from a “starting strain” that can be chosen by the operator or defined automatically as the most central of the distribution using the algorithms developed elsewhere [36], based on the evidence that the most central individual of a distribution is the one with the lowest average distance calculated from a distance matrix including all members of the distribution. Once strains are ordered, *HomoDist* generates the first tree with the starting strain and the three closest strains, calculating:

- *disCen*. refers to the distance of the elements of the series from the central strain.
- *Maxd*. represents the maximum distance of the sequences in the alignment.
- *NJtree*. is the tree obtained using the neighbour joining clustering method.
- *Utree*. is the tree calculated with the algorithm UPGMA.
- *CI*. is the consistency index, defined as the minimum number of changes divided by the number of changes required on the tree.
- *Retention Index.* measures the fraction of apparent synapomorphy to actual synapomorphy.
- *HI* is the Homoplasy index, the complement to 1 of *CI*.
- *SH* is an index that correlates homoplasy to distances. It is calculated as

$$\frac{HI}{MaxD}$$

Then, the successive strain in order of increasing distance (calculated applying the evolutionary model “F84” to *ITS* and *LSU* alignment) is added, another tree is built to calculate the parameters described above and so on until the completion of the strain series taken from a FASTA file alignment, used as the sole input of the algorithm.

The command to call the function was: *homo (“input.fas”, arguments).* The arguments were the following:

- *autCen*. can be set as True or False. When True, the algorithm searches for the absolute center of the distribution, which is the object minimizing the distances with all other members of the set. On the contrary, the option False allows the user to choose the center of the series.
- *defCen* defines the position, in the alignment, of the sequence chosen as center when *autCen* is set as “False”.
- *distmodel*. defines the evolutionary model to be used. The default model is “F84” [37]

Note that homoplasy can be evaluated as HI or as CI. The two metrics are calculated as HI = 1-CI. In the first part of the article, CI is employed as usual to check the quality of the tree. In the second part of the paper, we used HI being the direct measures of homoplasy. In other words, CI and HI were used respectively as measure of the phylogenetic noise and as proposal of homoplasy as an auxiliary criterion for species separation.

### 2.4. Data Analysis

*HomoDist* was used to calculate indexes for the four taxonomic model under test, both at the species and strain level. For assessing the variation of homoplasy related indexes with the topology of the tree, we carried out the analyses considering different centers of the distribution. Data were then exported and analyzed in MS Excel.

## 3. Results

### 3.1. Consistency Index Decreases with the Increase of the Taxonomic Complexity

**Figure 1:**
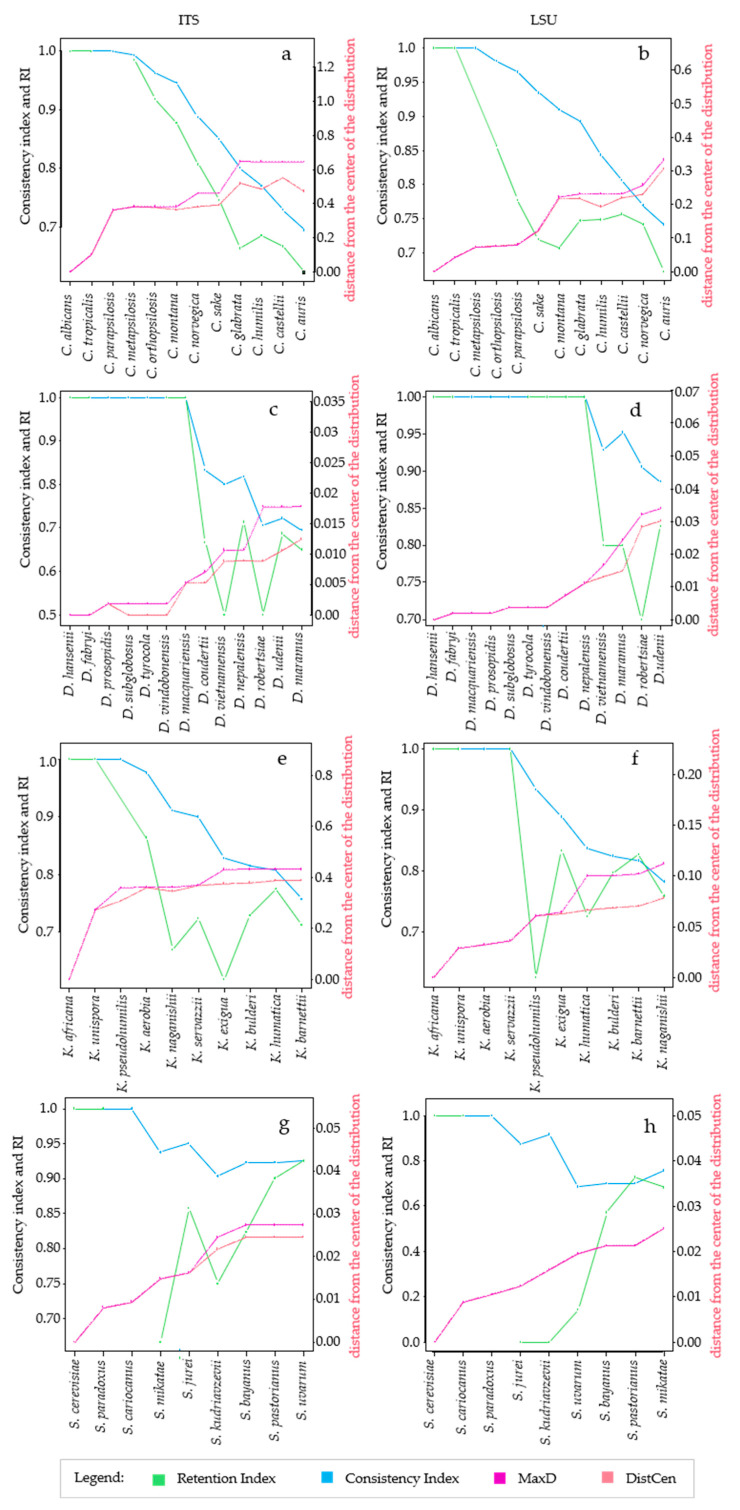
Variation of Homoplasy and phenetic Distances within different clades, at species level. The figure compares the trends of CI, RI and distances, taking into account one species at a time. Figure shows the trend of the metrics calculated for *ITS*, on the left, and *LSU*, panels on the right. The species considered for the analysis are reported in the x-axis, the leftmost species is the center of distribution. The left y-axis, in each plot, is a scale for Consistency and Retention Index and the secondary y-axis, on the right, is a scale for the distances. For every species added to the distribution, the algorithm calculates the following metrics: *MaxD*, in purple line, is the maximum distance obtained from the alignment of species. *DisCen*, in red line, is the distance of one species from the center of the series, which is represented by the first element in the X-axis. *Consistency index (CI)*, in blue line, is the ratio between the minimum number of changes it might show and the number of changes it does show on a particular tree. *Retention index*, represented with the green line, measures the fraction of apparent synapomorphy to actual synapomorphy. All these values were calculated starting from the alignment of *ITS* and *LSU* sequences of species within *Candida* clade, respectively (**a**,**b**); *Debaryomyces* clade, (**c**,**d**); *Kazachstania* clade, (**e**,**f**); *Saccharomyes* clade, (**g**,**h**).

It is well known from the early days of cladistics that the homoplasy tends to increase with the increase of the tree complexity, even with random data [12,14]. There is still no quantification of this phenomenon in microorganisms in which the homoplasy can be due to horizontal gene transfer and there is no quantification for rRNA markers widely used in taxonomy and phylogeny [26,28]. In this paper, we took into consideration different taxonomic models and analyzed them with *HomoDist* using the rRNA markers *ITS* and *LSU*, reporting the CI, that is the complement to 1 of HI, since CI is a direct measure of the tree quality. The application of this algorithm clearly showed the opposite trend of CI and RI vs. the distances (Figure 1), in the trees obtained by using the type strains of four groups of species belonging to the genera *Candida* (prevalently pathogenic species), *Saccharomyces, Kasachstania* and *Debaryomyces* (Figure S1).

In the group of species of the genus *Candida*, both *ITS* an *LSU* had similar behavior with a CI and RI ranging from 0.6 to 0.75 with distances between 30% to 60%. In both cases, CI had a rather monotonous decrease, whereas the RI could not be computed for some strains combinations and showed a change in trend with *LSU* (Figure 1a,b). The same features of CI and RI could be found in all taxonomic groups analyzed (Figure 1c through h) leading to the conclusion that the former is more amenable for this type of approach, although CI normally overestimates the consistency, in comparison to the RI, and therefore underestimates the homoplasy. Interestingly, the distance plot showed sudden increases, for example between *C. tropicalis* and *C. parapsilosis* or between *C. sake* and *C. glabrata* with *ITS* (Figure 1a); on the contrary, the CI decrease did not show sudden changes of the slope in the *Candida* group. Nine species of *Debaryomyces* (from *D. hansenii* to *D. nepalensis*) showed no homoplasy (CI = 1) with *ITS* and seven (from *D. hansenii* to *D. macquariensis*) with *LSU* (Figure 1c,d). With the selected species of the genus *Kazachstania*, the CI decreased smoothly when *ITS* was used (Figure 1e), whereas with *LSU* there was not any change in a group of species including *K. africana, K. unispora, K. aerobia* and *K. servazzi*, then it decreased when the other species were added (Figure 1f). Interestingly, when the CI remained constant, the distance increased from 0 to around 4%, which is four times the allotted threshold for *LSU* [28]. Finally, with the species of the *Saccharomyces* genus sudden slope changes occurred between *S. cariocanus* and *S. mikatae* (*ITS*, Figure 1g) and between *S. kudriazevii* and a group of species including *S. bayanus, S. pastorianus* and *S. uvarum* (LSU Figure 1h). It is interesting to note that there were no important slope changes of the CI within phylogenetically close groups of *Saccharomyces* species at the genomic level [22].

### 3.2. Consistency Index Decreases Linearly with the Increase of the Taxonomic Distances

**Figure 2:**
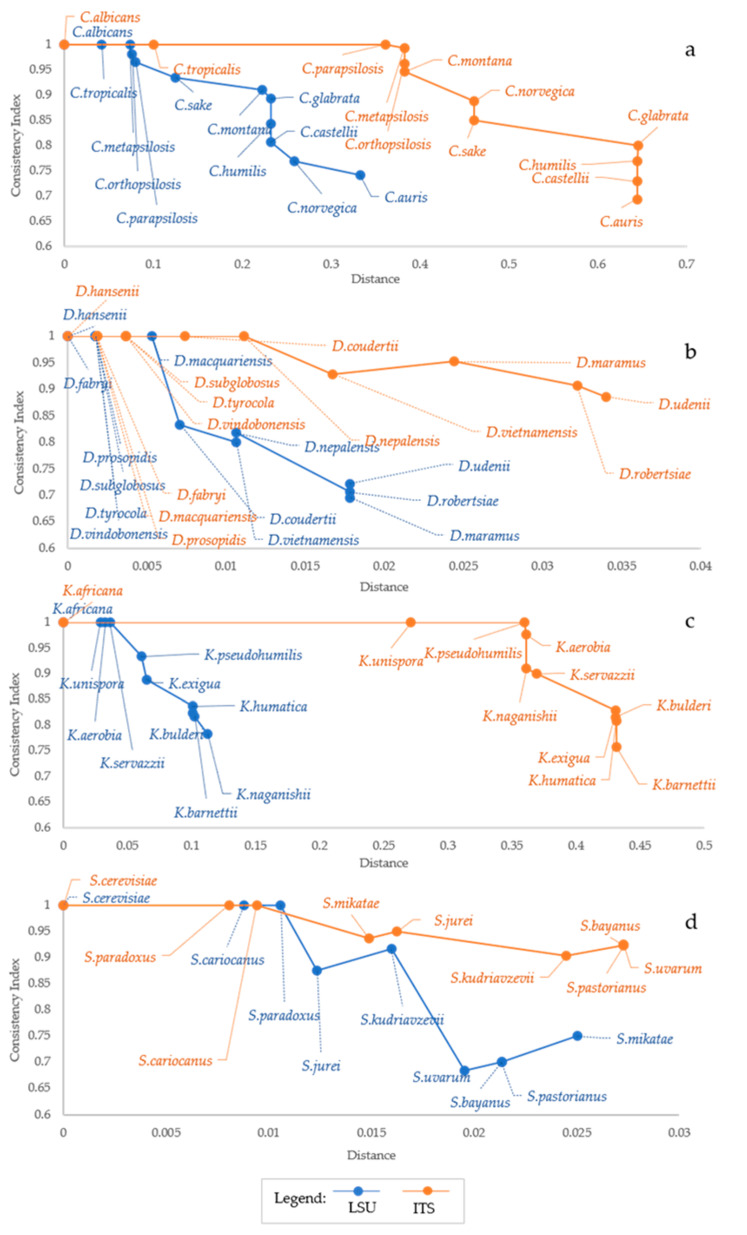
Relation between Distance and Consistency Index (CI). The plot shows the Consistency Index as a function of the distance (F84 distance model). Orange line represents data obtained from *ITS* sequence alignments while blue line is for *LSU* data. The figure highlights that CI (y axis) decreases with the increase of both distance (x-axis) and the number of species involved in the analysis (annotated directly on the figure). CI vs. distances trends of *Candida, Debaryomyces, Kazachstania* and *Saccharomyces* are reported in (**a**), (**b**), (**c**) and (**d**) respectively. Generally, CI values start decreasing with the fourth species added, only with *Debaryomyces,* it happened with the 7th and 9th species for *LSU* and *ITS*, respectively.

The results presented above show that CI decreases when more species are considered, although the addition of similar species does not produce any increase of homoplasy or CI decline. The species with no homoplasy displayed a maximum distance of 1% and 0.5% with *ITS* and *LSU*, indicating that the relationship between homoplasy and distance is not universal and depends on both the marker used and the taxa analyzed. These observations pose the question about the relation between homoplasy and taxonomic distance and on the different performances of the two markers used. When using *LSU*, CI decreased more rapidly than with *ITS* (Figure 2), although with different trends.

This was due also to the fact that *LSU* showed smaller distance ranges, whereas the decrease of the CI was nearly similar in most instances. In the *Candida* model, the descending trend of the CI was quite similar, although at different distances. Two strong decrease were observed at ca 8% and 23% distance from *C. albicans* for *LSU* and at 38% and 46% with *ITS* (Figure 2a). *Debaryomyces* showed only a sharp decrease of CI at less than 1% distance from *D. hansenii*, whereas CI from *LSU* decreased smoothly (Figure 2b). *Kazachstania* showed a rapid decrease of the CI from *LSU* at distances below 15%, whereas the CI from *ITS* showed a marked decrease only at over 35% (Figure 2b). Finally, the CI from *ITS* decreased smoothly in *Saccharomyces*, whereas that from LSU showed a marked decrease from *S. paradoxus* to *S. jurei* and from *S. kudriazevii* and the group *S. bayanus, S. pastorianus* and *S. uvarum* (Figure 2d). These observations indicate that the CI does not show sharp decreases corresponding to defined species limits, except in a few cases. Generally, the *LSU* distances are approximately half of the corresponding *ITS* distances, whereas the CI decrease is similar in all taxonomic group considered. A relatively high and negative correlation was found between CI and the maximum distance between species (Table 2).

**Figure 3:**
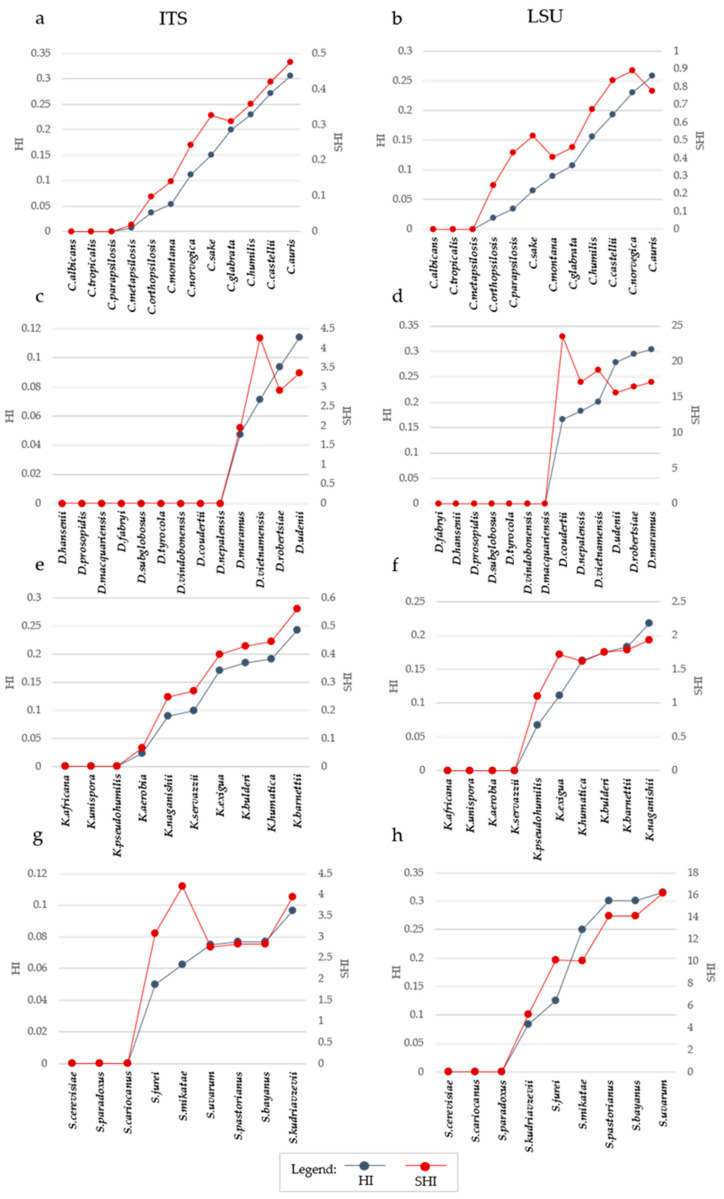
Trend of Homoplasy Index (HI) and Specific Homoplasy Index (SHI) with the increase of distance and number of species. Each plot reported in the primary Y-Axis (on the left) the value of HI, while the secondary Y-Axis (on the right) reported the value of SHI. In the X-Axis species are reported. Species are displayed in increasing distance order from the center, represented by the leftmost species of each graph. The plot shows the increase of homoplasy index (blue line) influenced by both the addition of strains to the series and the increase of distances. The red line shows the trend of the SHI. It is calculated as the ratio between HI and MaxD, which is the maximum distance of the alignment. (**a**,**b**) show the trend for *Candida ITS* and *LSU*, respectively. While panels (**c**,**d**) represent the trend for *Debaryomyces ITS* and *LSU*, respectively. (**e**,**f**) shows the trend for *Kazachstania ITS* and *LSU*, respectively. While (**g**,**h**) represent the trend for *Saccharomyces ITS* and *LSU*, respectively. SHI index results from the ration of HI and the corresponding distances, and therefore is a sort of Homoplasy normalized by the distance.

In the *Candida* model, characterized by higher inter-specific distances, the correlation was −0.944 and −0.910 for *ITS* and *LSU*, respectively. Not surprisingly, the other model with high correlations (−0.914 and −0.983 for *ITS* and *LSU*) was *Kazachstania* i.e., the other model with relatively high inter-specific distances. *Saccharomyces* and *Debaryomyces* showed more correlation at the *LSU* level (−0.730 and −0.978) than using *ITS* (−0.696 and −0.710). In order to visualize distances and homoplasy index (HI = 1 − CI) in a single graph, we reported the distance, the HI and the ratio between these two metrics, hereinafter referred to as SHI (Specific Homoplasy Index). The trends in Figure 3 showed that SHI increases with the distance, with few cases of peaks, as in *Debaryomyces* with both *ITS* and *LSU* (Figure 3b,c) and in *Saccharomyces* with *ITS* (Figure 3g).

SHI has not been designed to propose yet another threshold, but its trend (Figure 3) can be used to define when the tree has a strong increase of HI even by adding a few more taxa. In this sense, it is possible to consider SHI as an auxiliary tool in defining the size and complexity of the dendrograms.

### 3.3. Consistency Index Changes according to the Combination of Strain Considered

**Figure 4:**
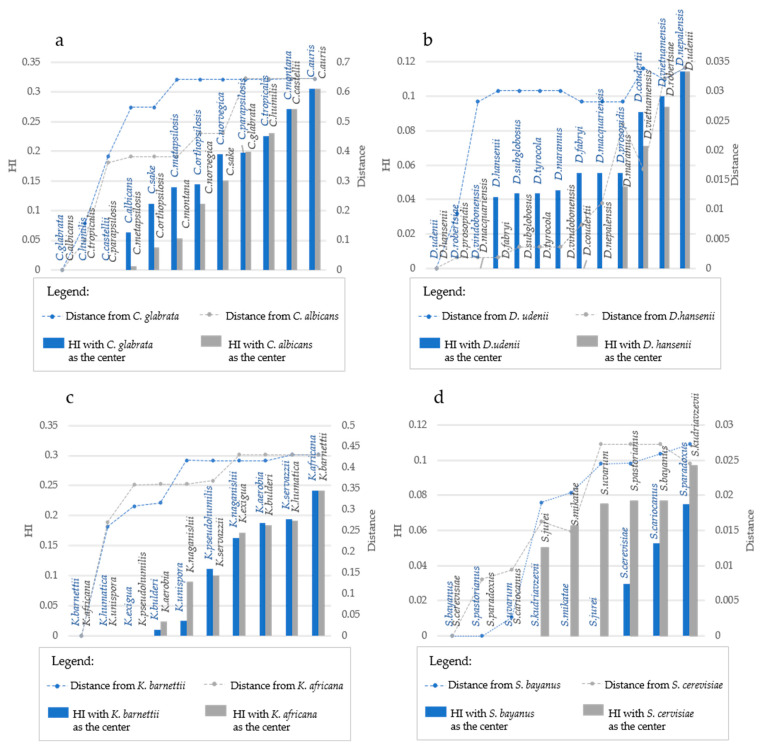
Homoplasy Index and Distance considering different starting strains. The plots report the variation in Homoplasy index and distance of *ITS* sequences, when two different species are considered the center of the distribution. Each plot reported in the primary Y-Axis (on the left) the value of HI, while the secondary Y-Axis (on the right) reported the value of distance from the center of the distribution. In the X-Axis species are reported. Species are displayed in increasing distance order from the center, represented by the two leftmost species of each graph. In (**a**), the gray bars represent homoplasy index and the gray line shows the trend of distance when *C. albicans* is considered the center, while blue bars and line represent respectively HI and distance when *C. glabrata* is the center of the distribution. In (**b**), gray bars represent homoplasy index and the gray line shows the trend of distance when *D. hansenii* is considered the center, while blue bars and line represent respectively HI and distance when *D. udenii* is the center of the distribution. In (**c**), gray bars represent homoplasy index and gray line shows the trend of distance when *K. africana* is considered the center, while blue bars and line represent respectively HI and distance when *K. barnettii* is the center of the distribution. In (**d**), gray bars represent homoplasy index and gray line shows the trend of distance when *S. cerevisiae* is considered the center, while blue bars and line represent respectively HI and distance when S. *bayanus* is the center of the distribution. The figure displays a difference in HI increase depending on the genetic distance. Genera composed of highly divergent species, such as *Candida* (**a**) and *Kazachstania* (**c**), did not show trend variation with different starting taxa.

The algorithm proposed to evaluate the evolution of the Consistency Index starts with a taxon (i.e., a species or a strain) and then proceeds aggregating more taxa in order of distance starting from the closest. It is therefore clear that the initial taxon plays a role in the CI analysis. To elucidate this aspect, in each of the four taxonomic models, we performed the analyses of the *ITS* described above, changing the initial species. The comparison between two analyses with different initial strains showed a totally different trend at the short and intermediate distances, whereas at long distances, the HI is practically identical in the two analyses (Figure 4).

More specifically, using *C. albicans* as center, the HI started increasing with distances over 30%, whereas with *C. glabrata* the distance at which the HI appeared was >50% (Figure 4a). *Debaryomyces* is a genus characterized by some close species, and the detection of the HI occurred at 2.4% distance starting with *D. hansenii* and 3% when the origin was *C. udenii* (Figure 4b). Similarly, in *Kazachstania*, the HI was detected at 31% and at 36% distance with *K. barnettii* and *K. africana* as initial species, respectively (Figure 4c). On the contrary, in *Saccharomyces* there was a large difference due to the initial strain; in fact, homoplasy was found at 2.45% and 1.62% distance when *S. cerevisiae* and *S. bayanus* were respectively used as starter strains (Figure 4d). These figures indicate that the HI starts increasing when the distance increases, but it depends also on the combination of species used. The different distances at which homoplasy started appearing in various models could be due to the average distance among the analyzed species, which was very low in *Debaryomyces* and *Saccharomyces* models and larger in the two other species panels analyzed.

### 3.4. Homoplasy Variations Using more Strains per Species

Taking advantage of the evidence that CI decline depends on the amount and distance of the taxa analyzed, we decided to deepen the analysis by constructing the trees with more strains per species and not only the type (Figure S2).

**Figure 5:**
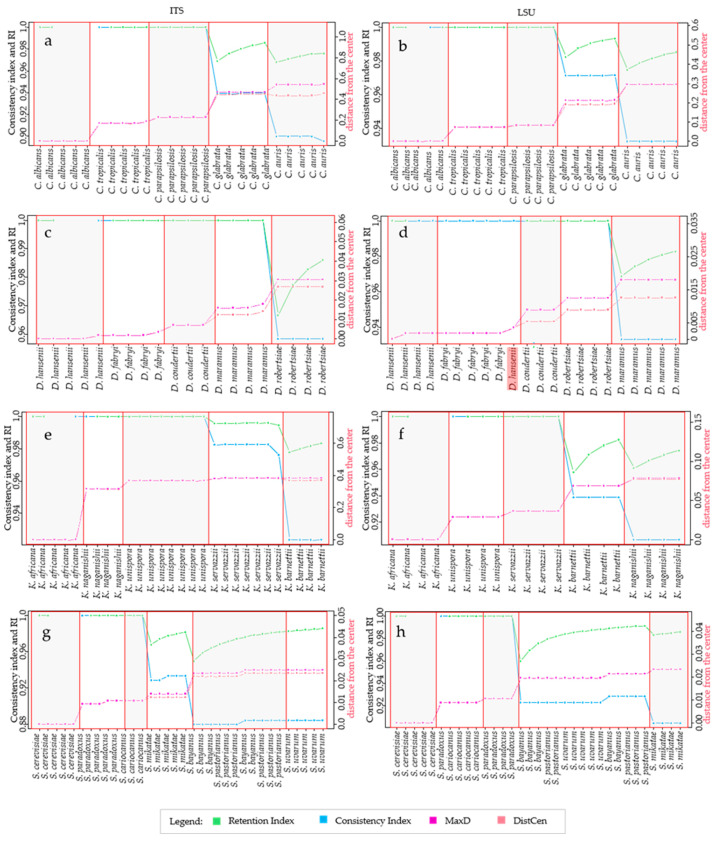
Variation of Homoplasy and phenetic Distances within different clades, at strain level. For every strain added to the series, the algorithm calculates the following metrics: *MaxD* (purple line), *DisCen* (red line), *Consistency index* (blue line), *Retention index* (green line). All these values were calculated considering different strains for every species included in the analysis. Plots show metrics obtained from *ITS* and *LSU* sequence alignment of different strains within *Candida* clade, respectively in (**a**,**b**); *ITS* and *LSU* data from strains of *Debaryomyces* in (**c**,**d**); *Kazachstania*, panels (**e**,**f**); *Saccharomyes* clade, (**g**,**h**). In general, CI is stable at its maximum = 1 with three species. Upon addiction of the first strain of the fourth species, CI decreases and remain stable until strains of the fifth and following species are added.

The analysis of the *Candida* model with both markers showed that the CI and RI trend presented two decrease subdividing the species in three groups: one with *C. albicans, C. tropicalis* and *C. parapsilosis*, another with *C. glabrata* and the last with *C. auris* (Figure 5a).

Interestingly, there were no changes of CI within these groups, whereas RI showed an increase within the second and third group. The distances increased gradually between each species, whereas the CI and RI decrease were very sharp in both cases. In *Debaryomyces*, the CI drop obtained with *ITS* and *LSU* separated respectively *D. robertsiae* and *D. marasmus* from all the other considered species (Figure 5c,d). *K. barnetti* was split from the other according to CI and RI from *ITS*, whereas *LSU* produced two drops that discriminated *K. barnetti* and *K. naganishi* from the rest of the species (Figure 5e,f). *Saccharomyces* showed two CI and RI drops with both markers producing the separation of three groups, one containing *S. cerevisiae*, *S. paradoxus* and *S. cariocanus*, a second with *S. mikatae* and a third with *S. bayanus, S. uvarum* and *S. pastorianus* (Figure 5f,g).

### 3.5. Using homoplasy for Species Delimitation

The analysis of homoplasy trend with more strains per species was tested with both *ITS* and *LSU*. The rationale is that a limited amount of homoplasy should be accumulated within the same species, although the idiosyncrasies of the HI suggest that this can be more visible when the species to test are at relatively high distance from the first taxon used to start the algorithm.

**Figure 6:**
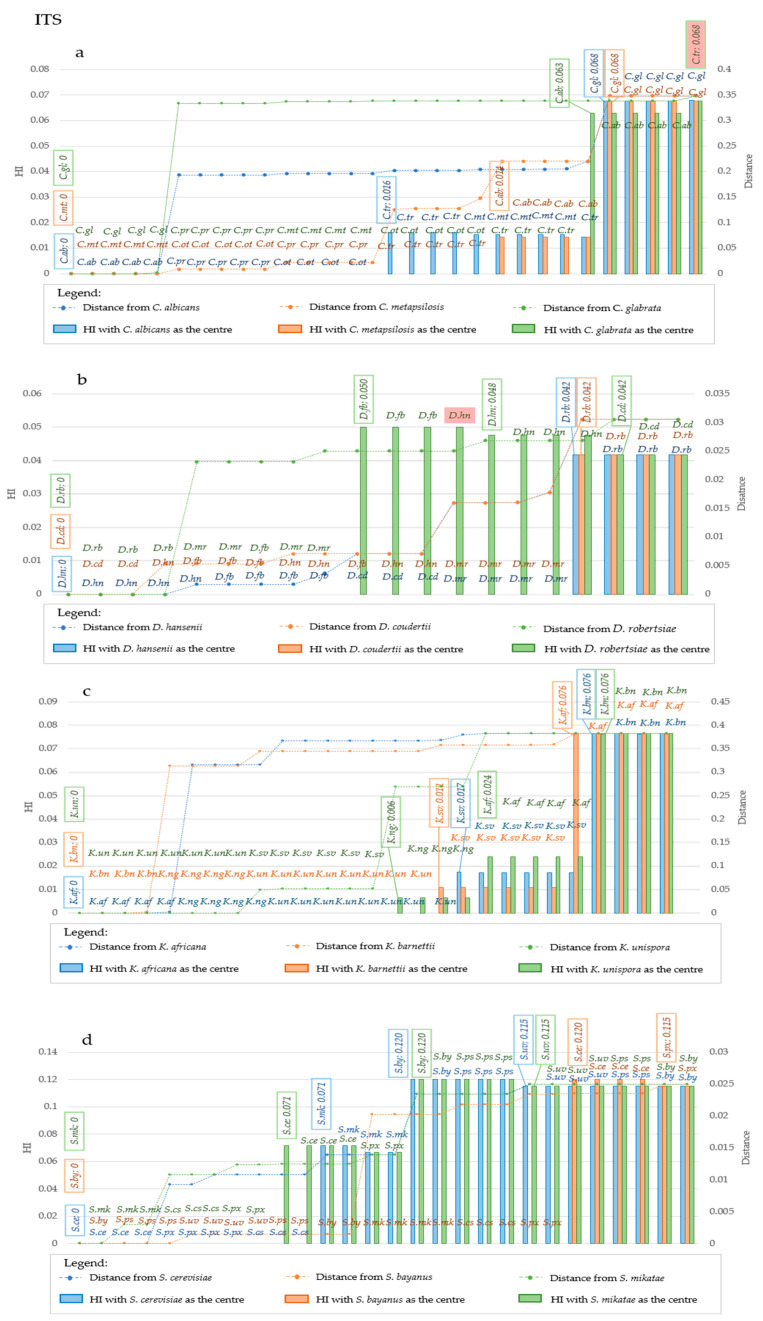
Homoplasy Index and Distance in *ITS* sequences considering different center of distribution, at strain level. The plots report the variation in Homoplasy index and distance of *ITS* sequences, when three different species are considered the center of the distribution. Each plot reported in the primary Y-Axis (on the left) the value of HI, while the secondary Y-Axis (on the right) reported the value of distance from the center of the distribution. In the X-Axis species are reported. Species are displayed in increasing distance order from the center, represented by the three leftmost species of each graph. In (**a**), blue bars and line represent respectively homoplasy index and distance when *C. albicans* is considered the center of the distribution, while orange bars and line represent respectively HI and distance when *C. metapsilosis* is the center, and green bars and line represent respectively Hi and Distance when *C. glabrata* is chosen as the central point. In (**b**), blue bars and line represent respectively homoplasy index and distance when *D.hansenii* is considered the center of the distribution, while orange bars and line represent respectively HI and distance when *D. coudertii* is the center, and green bars and line represent respectively Hi and Distance when *D. robertsiae* is chosen as the central point. In (**c**), blue bars and line represent respectively homoplasy index and distance when *K. africana* is considered the center of the distribution, while orange bars and line represent respectively HI and distance when *K. barnettii* is the center, and green bars and line represent respectively Hi and Distance when *K. unispora* is chosen as central point. In (**d**), blue bars and line represent respectively homoplasy index and distance when *S. cerevisiae* is considered the center of the distribution, while orange bars and line represent respectively HI and distance when *S. bayanus* is the center, and green bars and line represent respectively Hi and Distance when *S. mikatae* is chosen as the central point. Abbreviations on the same line indicate strains that are characterized by the same value of HI. Changes of HI are displayed by the explication of the HI value within the box, together with the strain in which such variation occurred. Such boxes are reported above the corresponding bar. Generally, these variations occur at the transition point between two species. Legend for the abbreviations: C. ab is for *C. albicans,* C. gl is for *C. glabrata,* C. mt is for *C. metapsilosis,* C. ot is for *C. orthopsilosis,* C. pr is for *C. parapsilosis,* C. tr is for *C. tropicalis.* D. cd is for *D. coudertii,* D. fb is for *D. fabryi,* D. hn is for *D. hansenii,* D. mr is for *D. maramus,* D. rb is for *D. robertsiae.* K. af is for *K. africana,* K. bn is for *K. barnettii,* K. ng is for *K. naganishii,* K. sv is for *K. servazii,* K. un is for *K.unispora.* S. cs is for *S. cariocanus,* S. ce is for *S. cerevisiae,* S. mk is for *S. mikatae,* S. px is for *S. paradoxus,* S. ps is for *S. pastorianus,* S. uv is for *S. uvarum.*

The group *Candida,* analyzed using the *ITS* locus of *C. albicans* as starting taxon, did not show an increase of HI up to 1.6% distance, outlining three groups of which one included *C. albicans*, *C. parapsilosis* and *C. orthopsilosis*, whereas *C. tropicalis* and *C. metapsilosis* formed a second group, while the third is made of *C. glabrata* alone (blue series—Figure 6a).

**Figure 7:**
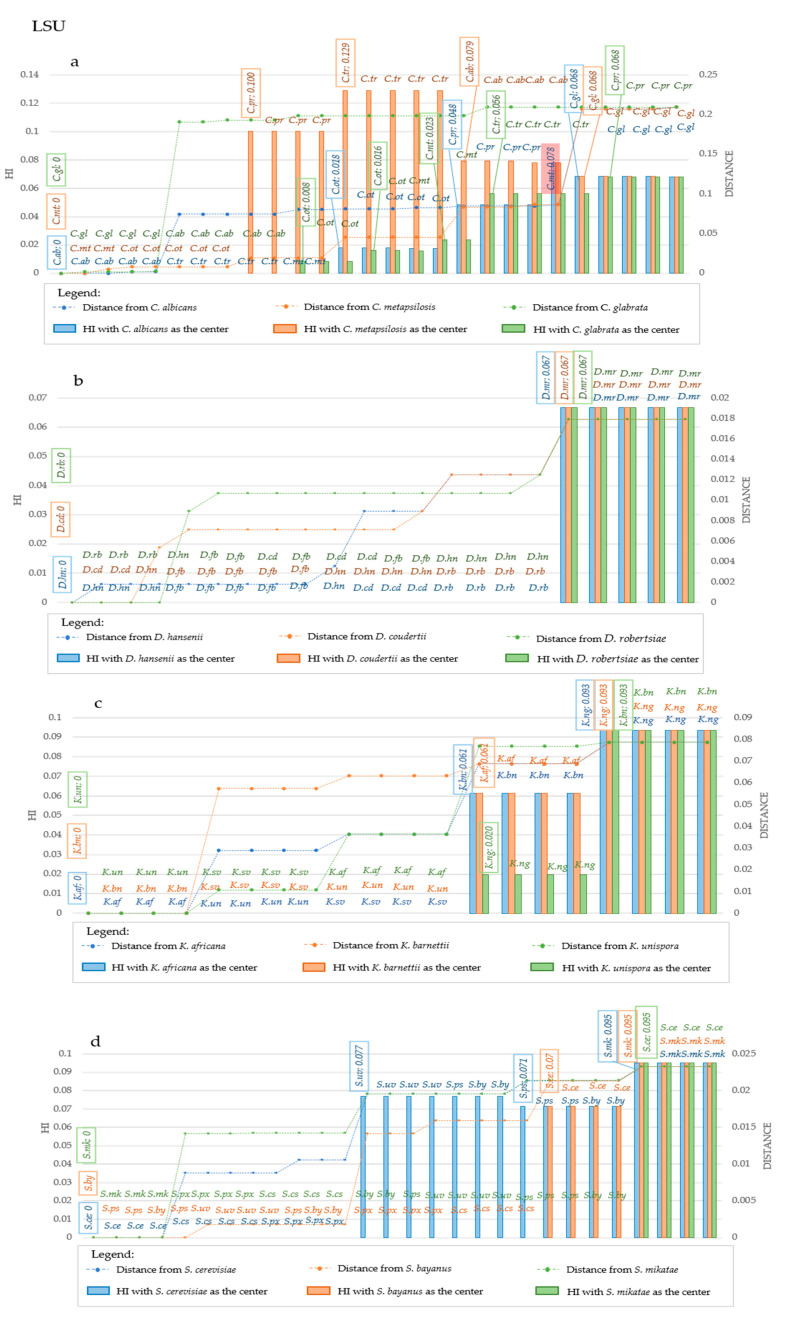
Homoplasy Index and Distance in *LSU* sequences considering different center of distribution, at strain level. The plots report the variation in Homoplasy index and distance of *LSU* sequences, when three different species are considered the center of the distribution. Each plot reported in the primary Y-Axis (on the left) the value of HI, while the secondary Y-Axis (on the right) reported the value of distance from the center of the distribution. In the X-Axis species are reported. Species are displayed in increasing distance order from the center, represented by the three leftmost species of each graph. In (**a**), blue bars and line represent respectively homoplasy index and distance when *C. albicans* is considered the center of the distribution, while orange bars and line represent respectively HI and distance when *C. metapsilosis* is the center, and green bars and line represent respectively Hi and Distance when *C. glabrata* is chosen as the central point. In (**b**), blue bars and line represent respectively homoplasy index and distance when *D. hansenii* is considered the center of the distribution, while orange bars and line represent respectively HI and distance when *D. coudertii* is the center, and green bars and line represent respectively Hi and Distance when *D. robertsiae* is chosen as the central point. In (**c**), blue bars and line represent respectively homoplasy index and distance when *K. africana* is considered the center of the distribution, while orange bars and line represent respectively HI and distance when *K. barnetii* is the center, and green bars and line represent respectively Hi and Distance when *K.unispora* is chosen as the central point. In (**d**), blue bars and line represent respectively homoplasy index and distance when *S. cerevisiae* is considered the center of the distribution, while orange bars and line represent respectively HI and distance when *S. bayanus* is the center, and green bars and line represent respectively Hi and Distance when *S. mikatae* is chosen as the central point. Abbreviations on the same line indicate strains that are characterized by the same value of HI. Changes of HI are displayed by the explication of the HI value within the box, together with the strain in which such variation occurred. Such boxes are reported above the corresponding bar. Generally, these variations occur at the transition point between two species. An exception to this observation is underlined with a red box in panel a, when a strain of *C. metapsilosis* was plotted within the group of *C. parapsilosis.* Legend for the abbreviations: C. ab is for *C. albicans,* C. gl is for *C. glabrata,* C. mt is for *C. metapsilosis,* C. ot is for *C. orthopsilosis,* C. pr is for *C. parapsilosis,* C. tr is for *C. tropicalis.* D.cd is for *D. coudertii,* D. fb is for *D. fabryi,* D. hn is for *D. hansenii,* D. mr is for *D. maramus,* D. rb is for *D. robertsiae.* K. af is for *K. africana,* K. bn is for *K. barnettii,* K. ng is for *K. naganishii,* K. sv is for *K. servazii,* K. un is for *K.unispora.* S. cs is for *S. cariocanus,* S. ce is for *S. cerevisiae,* S. mk is for *S. mikatae,* S. px is for *S. paradoxus,* S. ps is for *S. pastorianus,* S. uv is for *S. uvarum.*

A rather different grouping was observed using *LSU*: *C. albicans C. tropicalis* and *C. parapsilosis* formed the same group with distances up to 1.8%, whereas some strains of *C. parapsilosis* and all the *C. orthopsilosis* and *C. metapsilosis* fell in another group (blue series in Figure 7a).

Starting with *C. metapsilosis*, the HI from ITS allowed to group *C. metapsilosis, C. parapsilosis*, *C. orthopsilosis* and *C. tropicalis* in the same group, whereas *C. albicans* and *C. glabrata* formed two distinct clusters (Figure 6a orange series). The *LSU* derived HI grouped *C. metapsilosis* with *C. orthopsilosis,* then *C. parapsilosis* grouped in a specific group as *C. tropicalis* and *C. albicans* and *C. glabrata* (Figure 7a, orange series). Using *C. glabrata* as starting taxon, all species would be clustered together and *C. albicans* would be separated based on the *ITS*-HI, whereas with *LSU C. glabrata* and *C. albicans* clustered together, then all the others clustered in different groups in which there was little if any HI variability amongst members of the same species (Figure 6a and Figure 7a, green series). Using the same approach, the HI level produced two groupings with *LSU* and *ITS* in *Debaryomyces* (Figure 6b and Figure 7b). Only using *D. robertsiae* with *ITS* was possible to produce an intermediate group including *D. hansenii* and *D. fabryi* (Figure 6b, green series). In all cases studied, the HI did not discriminate between the starting taxon and the closest, up to distances around 4% for *ITS* and 6.7% with *LSU*. The assemblage in *Kazachstania* was more articulated; in fact, *ITS* produced 3, 3 and 4 groups with 1.7%, 1.1% and 2.4% distance within the first group, using as starting taxon respectively the type strains of *K. africana, K. barnettii* and *K. unispora* (Figure 6c), respectively. *LSU* always produced three clusters and the group closer to the starting taxon showed a maximum distance of 6.6% with *K. africana* and *K. barnetti*, whereas it was 2% with *K. naganishi* (Figure 7c). Considering ITS, *Saccharomyces* species formed two groups, with *S. cerevisiae* and *S. bayanus* as starting taxa, whereas with *S. mikatae* three groups were obtained. HI was detected at 7.1% distance from the starting strain in the first two cases and at 12% when starting with *S. mikatae* (Figure 6d). *LSU* produced four groups with *S. cerevisiae* as starting strain (7.7% distance), and two groups with the other two species as starting taxon (Figure 7d). The evidence that *S. bayanus*, *S. pastorianus* and *S. uvarum* could not be separated with the analysis of the HI, supports the actual efficacy of this method, since these three species are interconnected by intra-specific hybridizations [22]. On the contrary, this approach allowed the separation of two non-hybrid species such as *S. cerevisiae* and *S. paradoxus* using *LSU*-derived HI (orange series of Figure 7d) and partly also using *ITS* (orange series of Figure 6d), in both cases starting with the type of *S. bayanus.*

## 4. Discussion

Homoplasy limits somehow the accuracy of cladogram reconstructions [5] and has been proposed as a system to evaluate the interruption of gene flow between species, using whole genome analysis [6,38]. It was already demonstrated that it depends on a series of factors such as the addition of characters and, more importantly, the addition of taxa [14]. The rationale behind this paper was to explore the possibilities offered by the study of homoplasy in species delimitation, using four partial taxonomic models, characterized by different distances among species and different sexual or asexual reproduction systems. In fact, the *Candida* model only includes asexual species, whereas the other three after the “one fungus one name” revolution [39] can include both sexual and asexual species. Furthermore, the *Saccharomyces* model includes intra-specific hybrids [22], whereas *Debaryomyces* is characterized by species at close distances and with relative genetic homogeneity within populations, probably due to the possibility of some species to carry out conjugation even within the ascus [40,41,42]. The phylogenetic analysis was carried out using *ITS* and *LSU* because they are the most used taxonomic markers since the introduction of sequences as taxonomic tools [26,28]. Although the use of other single copy markers has been proposed [43] and proved to be even more effective than rRNA genes, their availability in public reference databases is still scarce, making their extensive use difficult [44].

In this paper, we could confirm that homoplasy increases, and correspondingly CI and RI decrease, when adding more taxa and that there is an almost linear relationship between HI and distance in some models, although this aspect cannot be generalized. Not only increases in homoplasy with taxa addition [14], but its variation depends also on which species are included in the phylogenetic analysis. These aspects caused homoplasy to become evident at higher distances among type strains of the species (Figure 3). Even when using more strains per species, it was impossible to detect a drop of the CI corresponding to the boundaries of the species analysed. These figures suggest that homoplasy calculated with the markers we used cannot be taken into consideration as a tool to discriminate species and their boundaries as suggested by genomic studies [6]. Even more importantly, whereas Bobay noted a decrease of homoplasy at the boundary between species, we observed the increase that was observed and predicted elsewhere [14,45]. On the other hand, the evidence that species barriers are “semiporous,” thus allowing extensive horizontal gene transfer suggests an increase rather than a decrease of homoplasious sites with the expansion of the genetic distance [16]. The differences from our results and those from genomic analyses, can be due to the different types of genetic information analyzed. Bobay and co-workers used protein coding genes, whereas our analysis had to be restricted to two short sequences of which one encodes a part of the large subunit ribosomal RNA and one is a spacer. The multi-copy nature of rRNA genes and their internal variability [30,31] make these markers quite different from protein encoding single copy genes, and therefore the results of these two model studies cannot be compared.

Although homoplasy is strongly considered the consequence of horizontal gene transfer, convergent evolution is an important, mostly underestimated, factor as well [46,47]. Whatever the nature of the homoplasy observed in rRNA markers, its trend suggests an accumulation of homoplasious sites at distances far larger than those proposed for species delimitation i.e., 1% and 1.4% for *LSU* and *ITS*, respectively [27,28], suggesting two main implications.

Firstly, homoplasy and horizontal gene transfer do not seem to imply less efficacy of phylogenetic reconstructions at relatively short distances, confirming a more generalized conclusion on the importance of bifurcating trees in bacterial phylogeny [3]. On the contrary, long-range and complex reconstruction should be carefully evaluated according to the first Hennig’s auxiliary principle that regulates the validity of the tree, according to the homoplasy found [48]. Since tree complexity is the other well-known factor implying high HI, trees with only type strains can be a solution to simplify the tree. Trees with only type strains are often used to describe new species [49], by necessity for the presence of a single strain in many species and the absence of no official reference of the species, although the “*International Code of Nomenclature for algae, fungi, and plants*” states that the type “*is not necessarily the most typical or representative element of a taxon.*” The data presented in this paper using only the type strains did not show generalizable trends of HI increase, equivalent to CI decrease, that can be used in the species delimitation, but only an evaluation of how the phylogenetic signal decreases with both complexity and distance among taxa. Taking into consideration both complexity and distance, the use of type strains in conjunction with the Specific Homoplasy Index (SHI) could be proposed to normalize the HI and evaluate the tree not with the homoplasy per se but rather with the homoplasy relative to the distance among the taxa in the tree. Furthermore, different SHI values indicate differences in the evolutionary traces included in the phylogenetic tree. This inhomogeneity of evolutionary rates and modes within the taxonomic space should be analyzed in more detail in the future to understand whether it derives from sampling defect, well in line with the estimate of known fungal diversity as a minority of the total [50], or whether it is due to other intrinsic aspects typical of different taxonomic groups. The second hypothesis open the further problem on whether species delimitation should follow the same rules with all groups (monism) or to be different in each group (pluralism), which definitely deserves more insight [24].

Secondly, the homoplasy did not show changes within the species, and not even within species close to the starting strain. It suggested that HI analysis could be used as an auxiliary tool in species delimitations, provided that the starting strain of *HomDist* is relatively far from the species to delimit. This means that close species can be dissected with this criterion when a relatively distant starting strain is chosen. The proposed algorithm *HomoDist* is designated to accumulated taxa beginning with a starting taxon and then adding other taxa one by one in order of increasing distance. In this way, the homoplasy trend can be studied at every addition. The fact that the homoplasy increases with the tree complexity, already stated in seminal and theoretical works [12,14], was confirmed in this paper. On the other hand, it implies that the sensitivity of the algorithm is low for taxa close to the starting taxon, whereas it increases with successive additions of other more distant taxa. Taking into consideration all these clues, the analysis of HI variation can be applied to the taxa under study using the type of a different and relatively close species as starting taxon. The examples presented in this paper suggest that there is not the need for a large distance between the species to be delimited and the starting species, although distances over 4% seem to be ideal. The starting strain, in some way, is the equivalent of the outgroup that is used in statistics and dendrogram construction. This system of species delimitation at the present is not proposed to generate species delimitations autonomously and automatically, but rather to give more support to other delimitation or species hypotheses [51]. It can be used in database curation along with accurate distance analysis.

## 5. Conclusions

The study of homoplasy in relation to the genetic distances among the taxa has been investigated with the two-fold purpose of defining the impact of this metric in actual phylogenetic reconstructions and to propose an auxiliary system to evaluate fungal species delimitations. Widely used markers such as *LSU* and *ITS* provided enough insight in these two aspects to suggest that homoplasy is not a serious problem in phylogenetic reconstructions, if the distances are relatively small, and the tree complexity is not overwhelming. In this respect, the specific homoplasy (SHI) can help in defining the tree complexity and in shedding more light on the phylogeny of the group under study. On the other hand, the small variation of HI within the species and its increase among species suggest that it could be used as auxiliary system for species delimitation, especially taking advantage of the homoplasy stability within the species. Whether this criterion can be actually applied to taxonomic diagnostics is matter of further works employing more strains per species, and possibly different markers, to gain more insight in this field that we tried to start exploring.

## Figures and Tables

**Table 1:** Genbank accession numbers of type strain sequences that were used for species level analysis.

	Species	ITS Sequences	LSU D1/D2 Sequences
***Candida***	*C. albicans*	AB032172	U45776
	*C. auris*	AB375772	AB375773
	*C. castelli*	AY046196	U69876
	*C. glabrata*	AY046165	U44808
	*C. humilis*	AY046174	U69878
	*C. metapsilosis*	FJ872019	AY497667
	*C. montana*	GU246257	U62305
	*C. norvegica*	NR_111209	U62299
	*C. orthopsilosis*	FJ872018	FJ746056
	*C. parapsilosis*	NR_130673	U45754
	*C. sake*	AJ549822	U45728
	*C. tropicalis*	AF287910	U45749
***Debaryomyces***	*D. coudertii*	AM992914	U45846
	*D. fabryi*	AB053098	U94927
	*D. hansenii*	AF210327	U45808
	*D. macquariensis*	AM992909	FR799729
	*D. maramus*	AB053102	U45838
	*D. nepalensis*	AB053099	U45839
	*D. prosopidis*	NR_077067	AB054993
	*D. robertsiae*	AB054019	U45805
	*D. subglobosus*	EU816232	EU816297
	*D. tyrocola*	EU816237	EU816302
	*D. udenii*	NR_077068	U45844
	*D. vietnamensis*	AM992908	AM992907
	*D. vindobonensis*	FN598876	FN598875
***Kazachstania***	*K. aerobia*	NR_077087	AY582127
	*K. africana*	AY046155	U68550
	*K. barnettii*	AY046173	U84231
	*K. bulderi*	AY046172	AF398486
	*K. exigua*	AY046170	U68553
	*K. humatica*	AB097397	AB040999
	*K. pseudohumilis*	2-FJ888526	FJ888526
	*K. naganishii*	AB097398	AB088404
	*K. servazzii*	AY046153	U68558
	*K. unispora*	AY046154	U68554
***Saccharomyces***	*S. bayanus*	AY046152	U94931
	*S. cariocanus*	AY046147	AF398478
	*S. cerevisiae*	AY046146	U44806
	*S. jurei*	HG764814	HG764813
	*S. kudriavzevii*	AY046150	AF398480
	*S. mikatae*	AY046149	AF398479
	*S. paradoxus*	AY046148	U68555
	*S. pastorianus*	AY046151	AY048172
	*S. uvarum*	AY130306	AY130339

**Table 2:** Correlation between CI and maximum distance between species. Correlations were calculated according to the Pearson moment using the distances and the relative CI values with CI < 1, i.e., for all values presenting some degree of homoplasy.

	ITS	LSU
*Candida*	−0.944	−0.910
*Saccharomyces*	−0.696	−0.730
*Kazachstania*	−0.914	−0.983
*Debaryomyces*	−0.710	−0.978

## Data Availability

No new data were created or analyzed in this study. Data sharing is not applicable to this article.

## Supplementary Materials

The following are available online at https://www.mdpi.com/2076-2607/9/2/273/s1. Table S1. GenBank accession numbers of organisms’ sequences that were used for strain level analysis. Figure S1. Tree obtained with neighbour joining algorithm, at species level. Panels a and b show tree obtained from *ITS* and *LSU* sequence respectively, considering only the type strain of the species included in the analysis for the genus *Candida*. Panels c and d show tree obtained from *ITS* and *LSU* sequence respectively, considering only the type strain of the species included in the analysis for the genus *Debaryomyces.* Panels e and f show tree obtained from *ITS* sequences and *LSU* sequence respectively, considering only the type strain of the species included in the analysis for the genus *Kazachstania.* Panels g and h show tree obtained from *ITS* sequences and *LSU* sequence respectively, considering only the type strain of the species included in the analysis for the genus *Saccharomyces.* Figure S2. Tree obtained with neighbour joining algorithm, at strain level. Panels a and b show tree obtained from *ITS* and *LSU* sequence respectively, considering different strains of the species included in the analysis for the genus *Candida.* Panels c and d show tree obtained from *ITS* and *LSU* sequence respectively, considering different strains of the species included in the analysis for the genus *Debaryomyces.* Panels e and f show tree obtained from *ITS* sequences and *LSU* sequence respectively, considering different strains of the species included in the analysis for the genus *Kazachstania.* Panels g and h show tree obtained from *ITS* sequences and *LSU* sequence respectively, considering different strains of the species included in the analysis for the genus *Candida.*
