# Supplementary figures and images for: Homoplasy as an Auxiliary Criterion for Species Delimitation
Source: Microorganisms. 2021 Jan 28;9(2):273. doi: 10.3390/microorganisms9020273 (PMC7911335; doi:10.3390/microorganisms9020273)

**
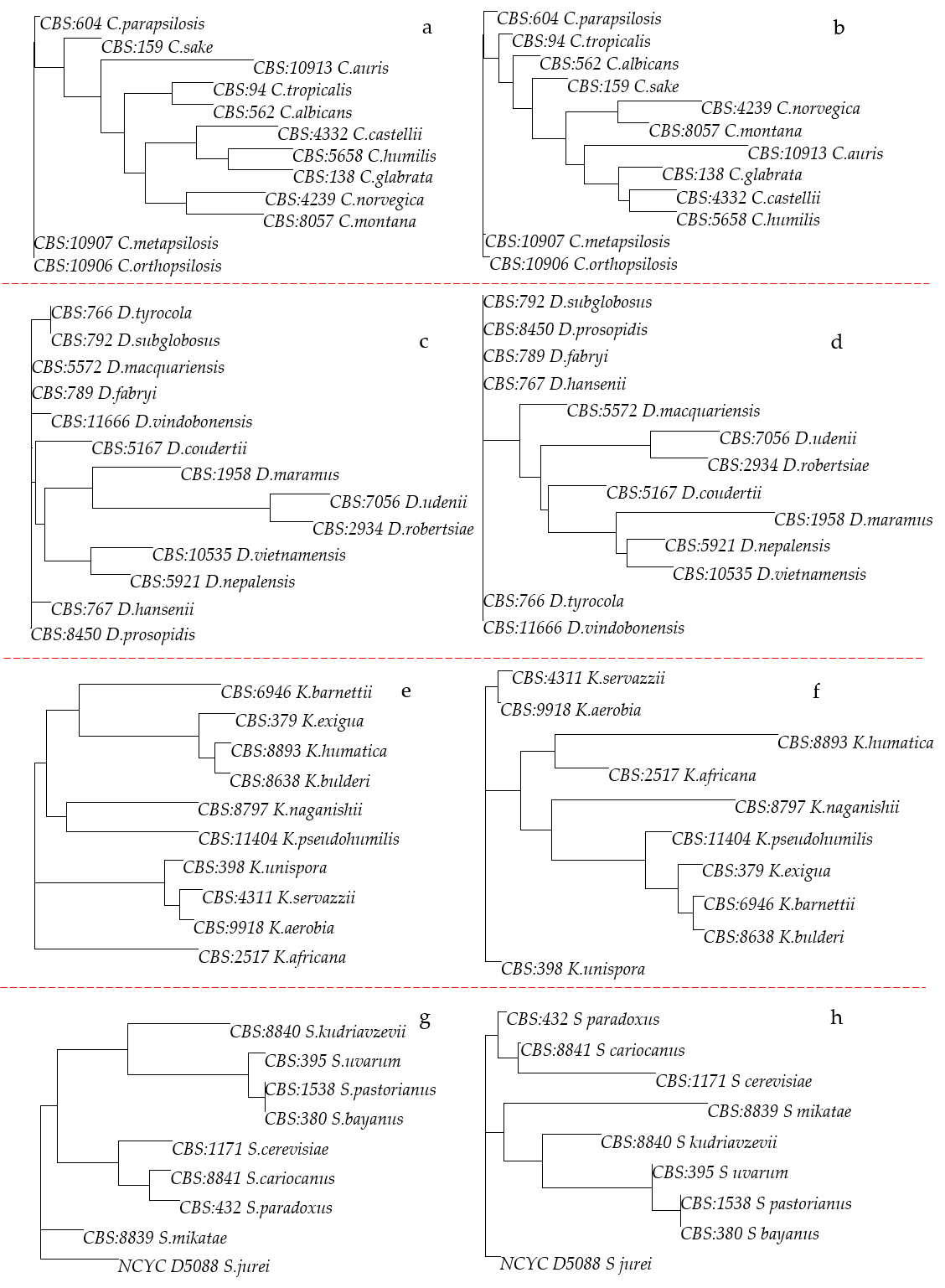
**

Supplement: Supplementary file 1 [file microorganisms-09-00273-s001.zip › Figure S1.docx]

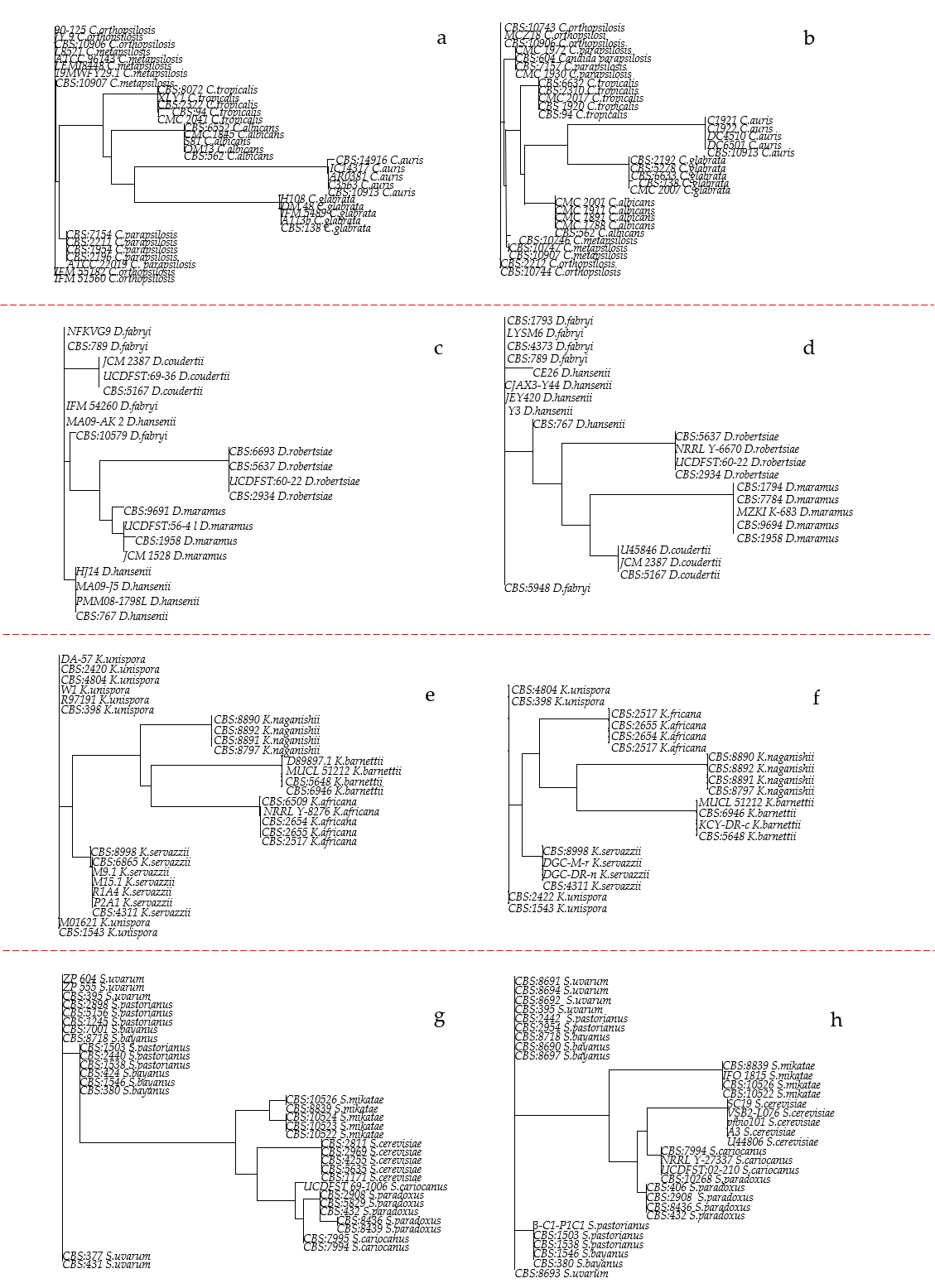

Supplement: Supplementary file 1 [file microorganisms-09-00273-s001.zip › Figure S2.docx]
